# BCL2-Rearrangment-Negative CD23+ Follicle Center Lymphoma and Chronic Lymphocytic Leukemia/Small Lymphocytic Lymphoma: A Rare Case of Biclonal Composite Lymphoma

**DOI:** 10.7759/cureus.88727

**Published:** 2025-07-25

**Authors:** Hira Qadir, Ejas Palathingal Bava, Juan Gomez-Gelvez, Wei Liu, Kedar Inamdar, Elizabeth Wey, John Carey, Yulei Shen, Philip Kuriakose, Sharmila Ghosh

**Affiliations:** 1 Pathology and Laboratory Medicine, Henry Ford Health System, Detroit, USA; 2 Hematology and Medical Oncology, Henry Ford Health System, Detroit, USA

**Keywords:** bcl2-r–negative, cd23-positive follicle center lymphoma, clonally distinct b-cell lymphomas, composite lymphoma, small lymphocytic lymphoma

## Abstract

Composite lymphomas are rare and diagnostically complex, comprising two or more distinct lymphoma subtypes within the same anatomical site and often requiring a comprehensive diagnostic approach. Due to their rarity and varied presentations, further research is needed to better understand their pathogenesis, clonal relationships, and optimal management strategies. This case report highlights an unusual presentation of composite lymphoma involving the coexistence of BCL2-R-negative, CD23-positive follicle center lymphoma and chronic lymphocytic leukemia/small lymphocytic lymphoma (CLL/SLL).

## Introduction

BCL2 rearrangement negative, CD23-positive follicle center lymphoma (BCL2-R-negative CD23+ FCL) is a rare, recently described provisional entity in the International Consensus Classification, 2022 [1]. While this entity is not formally included in the 5th edition of the WHO classification, it shares certain features with the diffuse variant of FL that has been described under the category of FL with unusual features [2]. This is a unique variant of follicular lymphoma characterized by some typical clinical and morphologic features such as involvement of inguinal lymph nodes, female predominate, a predominantly diffuse growth pattern, CD23 expression, lack of IGH::BCL2 rearrangement, 1p deletion, and stage I/II disease at presentation. The molecular profile includes a high frequency of *STAT6 and CREBBP *co-mutation as well as 1q gain and a recurrent 1p36 loss/TNFRS14 abnormalities [3,4]. The diagnosis of BCL2-R-negative, CD23+ follicle center lymphoma is challenging and must be differentiated from reactive conditions, nodal marginal zone lymphoma, and pediatric type follicular lymphoma. We report an unusually complex case of a composite lymphoma comprised of the recently described BCL2-R-negative, CD23+ follicle center lymphoma and a second low-grade B-cell lymphoma, which in this case was chronic lymphocytic leukemia/small lymphocytic lymphoma (CLL/SLL). The two B-cell lymphomas were clonally unrelated and arose from two distinct B-cell clones. The occurrence of composite lymphomas (CL) is infrequent, accounting for about 1.0% to 4.7% of all lymphoma cases [5,6], and composite lymphomas comprising two low-grade B-cell lymphomas are very rare [7]. These lymphomas may develop simultaneously or sequentially within a single anatomic site and can be either clonally related or unrelated [8]. Different combinations of composite lymphomas have been documented: (1) two types of non-Hodgkin lymphoma, usually B-lineage, (2) B-non-Hodgkin lymphoma and Hodgkin lymphoma, (3) two non-Hodgkin lymphoma of B and T lineage, (4) T-non-Hodgkin lymphoma and Hodgkin lymphoma. Composite lymphomas can be challenging to diagnose and can be missed on limited tissue samples, such as needle core biopsies. It underscores the role of advanced diagnostic modalities such as flow cytometry, fluorescence in situ hybridization (FISH), gene sequencing, and IGH gene rearrangement studies in the accurate diagnosis of such complex cases.

We present a rare case of composite lymphoma comprising two clonally distinct B-cell lymphomas, comprising CLL/SLL, and the recently described provisional entity of BCL2-R-negative CD23+ FCL involving multiple sites, including the parotid gland, cervical, and axillary lymph nodes. To the best of our knowledge, this has not been reported in the literature previously.

## Case presentation

**Figure 1:**
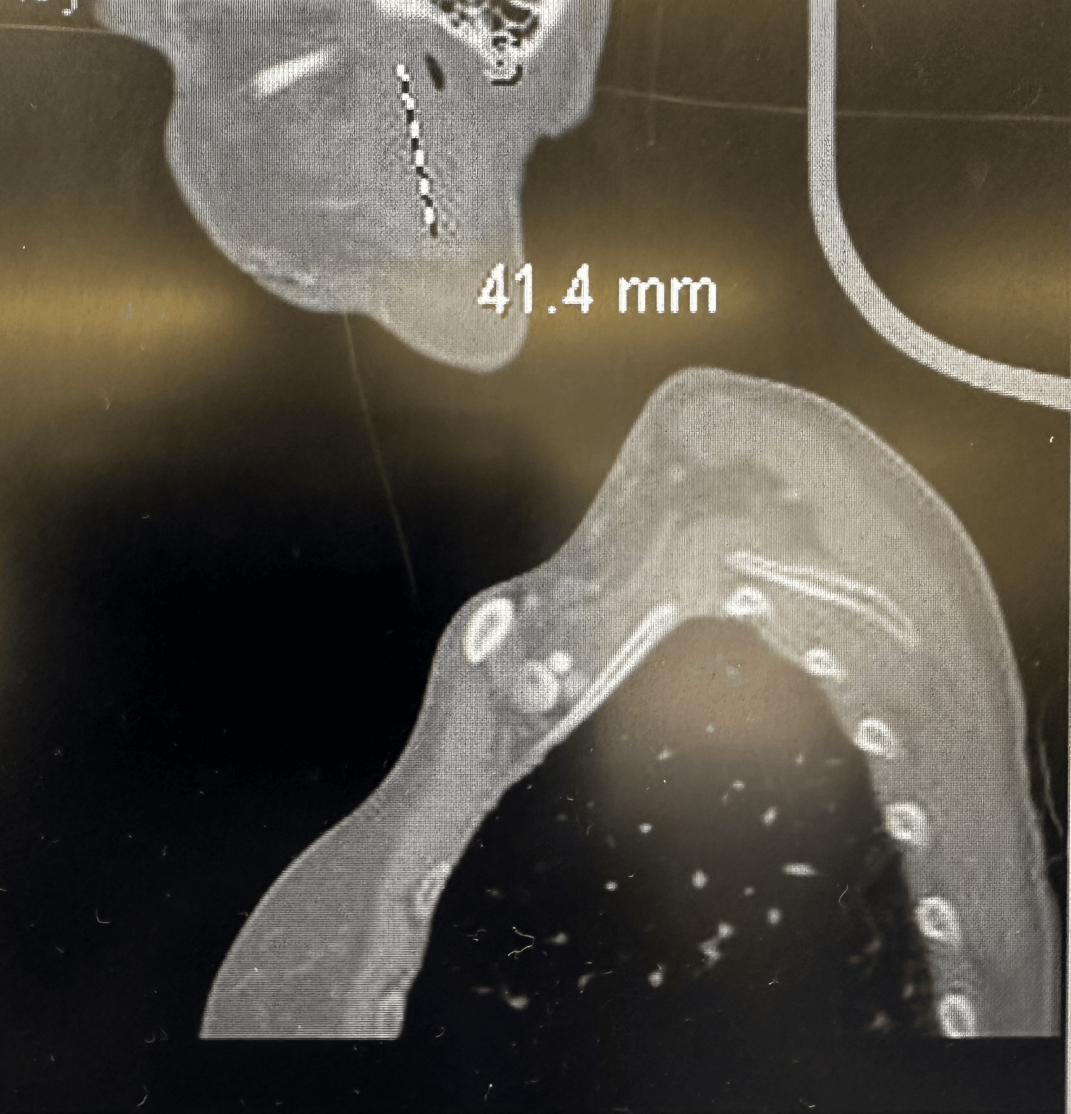
CT soft tissue neck CT soft tissue neck (sagittal view) showed right superficial parotid nodule measuring up to 4.1 cm

A 58-year-old female patient presented with fatigue and enlarged right-sided neck lymph nodes. CT revealed a 4.1 x 3.2 x 2.2 cm homogenously enhancing lesion of the superficial lobe of the right parotid gland and a 1.6 cm right level 5 lymph node, along with additional prominent nonenlarged jugular chain nodes (Figure 1).

**Figure 2:**
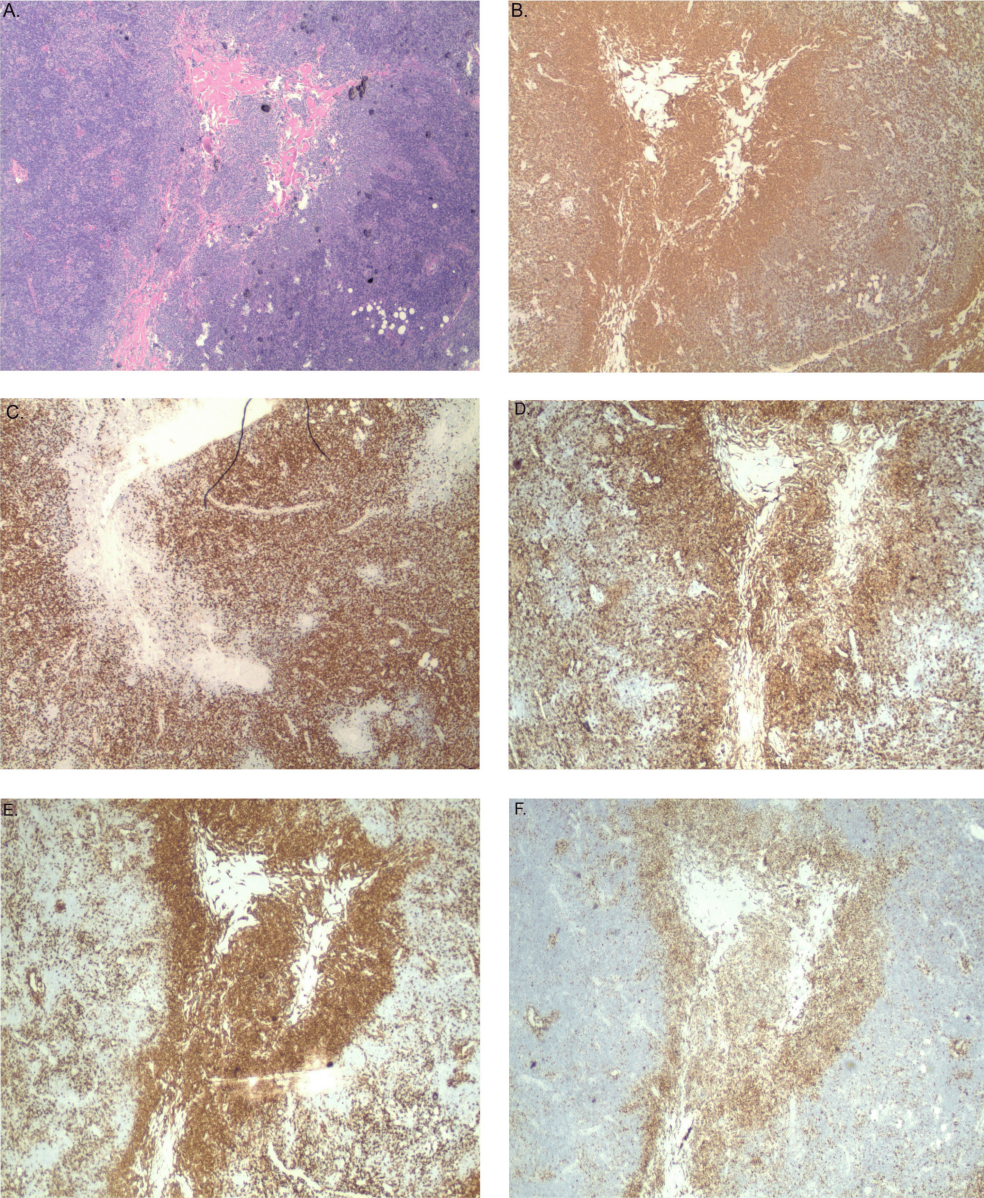
(A) H&E of BCL2-R-negative, CD23-positive follicle center lymphoma; (B) CD20; (C) CD3; (D) CD23; (E) CD10; (F) BCL-6 Atypical lymphoid infiltrate with centrocytic cells (A: H&E) showed positivity for CD20 (B), CD23 (D), CD10 (E), BCL-6 (F), and negative for CD3 (C) H&E: hematoxylin and eosin

A fine needle aspiration (FNA) of the cervical lymph node revealed an atypical lymphoid proliferation comprised of two monotypic B-cell populations identified by flow cytometry, one being CD5+ and the other CD10+. This was followed by an excision biopsy of a right axillary lymph node that revealed almost total nodal architectural effacement with a predominantly diffuse and vaguely nodular proliferation of small atypical lymphoid cells, microfollicle formation, and mild background sclerosis. A rim of normal lymphoid follicles was present at the periphery. The immunohistochemical staining of the atypical lymphoid infiltrate with centrocytic cells showed positivity for CD10, BCL6, and CD23, but was negative for CD5 and BCL2 (with two BCL2 antibody variants (clones 100/D5 and E17)), with a MIB1 proliferation index of 30%. A few follicles were highlighted by CD21+ residual follicular dendritic cell meshworks. The morphology and immunophenotype of these cells raised the possibility of BCL2-R-negative, CD23+ follicle center lymphoma (Figure 2).

**Figure 3:**
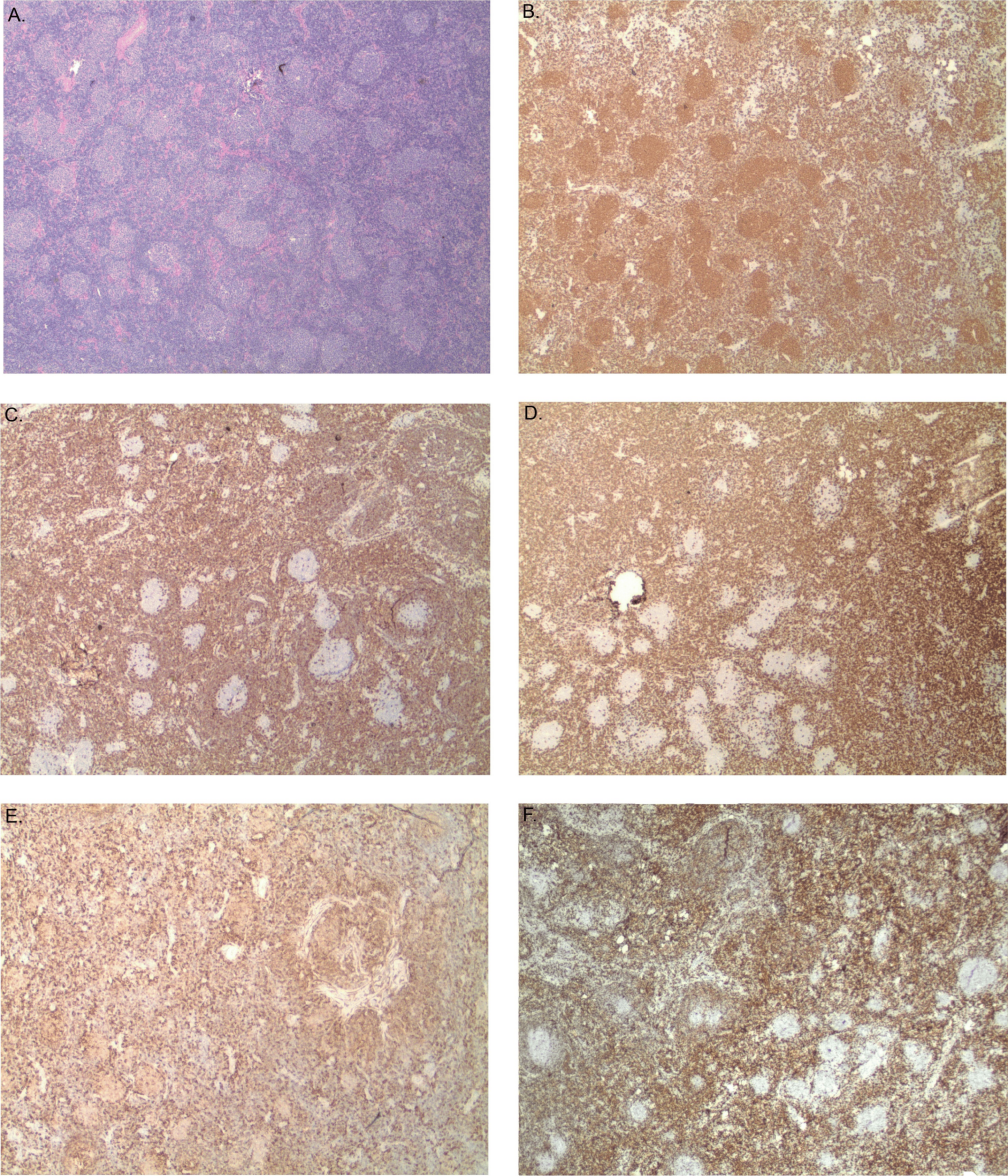
(A) H&E of CLL/SLL; (B) CD20; (C) CD5; (D) CD3; (E) CD23; (F) LEF-1 The small monotonous lymphocytes present in the interfollicular spaces (A: H&E) showed positivity for CD20 (B), CD5 (C), CD23(E), LEF-1 (F), and were negative for CD3 (D), compatible with CLL/SLL immunophenotype H&E: hematoxylin and eosin; CLL/SLL: chronic lymphocytic leukemia/small lymphocytic lymphoma

Additionally, a second subset of atypical lymphocytes, comprised of small lymphocytes with round nuclei and clumped chromatin, was also noted in interfollicular areas and raised the possibility of a second lymphoma likely to be chronic lymphocytic leukemia/small lymphocytic lymphoma (CLL/SLL). This was confirmed by the immunophenotype of this population of cells, characterized by expression of CD20 (dim), PAX5, CD5 (weak), CD23, BCL2, and LEF1, and negative for CD10, BCL6, BCL1, CD3, and SOX11. The MIB1 proliferation was 5-10% (Figure 3). SOX11 and BCL1 negativity ruled out mantle cell lymphoma (MCL). There was no peripheral blood lymphocytosis. Bone marrow biopsy showed normocellular marrow (30-40%) for age with minimal involvement (5%) by CLL/SLL based on flow cytometry and no evidence of follicular lymphoma.

FISH was performed on the right axillary lymph node with probes 1p36 (TP73) and 1q25 (ABL2), 3q27 (3'BCL6, 5'BCL6), 14q32 (IGH), 18q21 (BCL2). At least two hundred interphase cells were scored for each probe. The results were negative for 1p36 deletion, BCL6, and IGH::BCL2 gene rearrangements. Lymphoid neoplasm next-generation sequencing (NGS) panel performed on the lymph node showed STAT6 p.(Asp419Ala) VAF 5.4%, TNFRSF14 p.(Gln158*) VAF 5.4%, CREBBP p.(Ser1680del) VAF 8.1%, CREBBP p.(Asn984Ilefs*14) VAF 6.2%, FOXO1 p.(Met1?) start-loss, VAF 8% as variants of strong or potential clinical significance (Diagnostic, Prognostic & Therapeutic) TIER 1/2, which are commonly seen in association with the BCL2-R-negative, CD23+ follicle center lymphoma.

**Figure 4:**
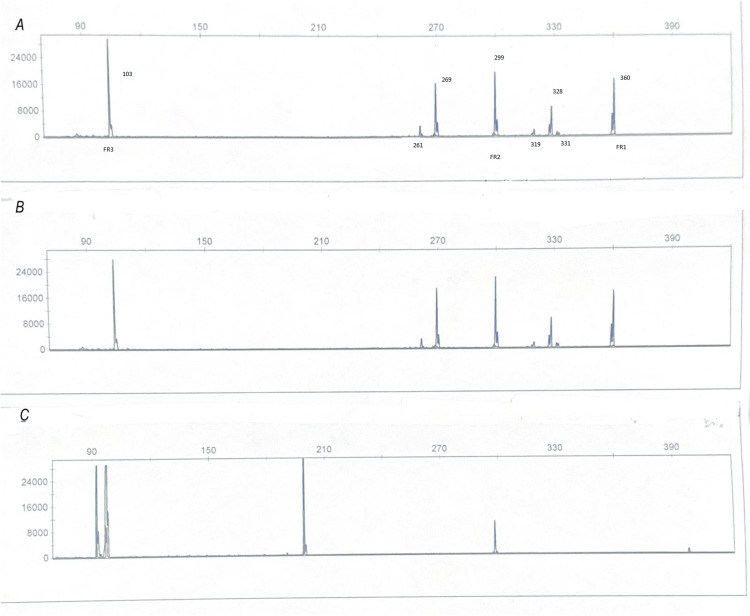
IGH gene rearrangement analysis IGH gene rearrangement analysis performed on the lymph node sample by polymerase chain reaction (PCR) showed two distinct peaks each in FR1 and FR2 regions, indicating a biclonal process (A) Patient sample; (B) patient sample duplicate; (C) specimen size control ladder

B-cell gene rearrangement studies by polymerase chain reaction (PCR) analysis were performed on the DNA obtained from the lymph node tissue and showed two clonal bands, a biallelic clone and a second clone, of distinctly different sizes (~360 bp and ~299 bp, respectively) in both frameworks 1 and 2 (Figure 4). The findings demonstrated that the two lymphomas had arisen from two separate B-cell clones, confirming the morphologic diagnosis of two separate B-cell lymphomas.

**Figure 5:**
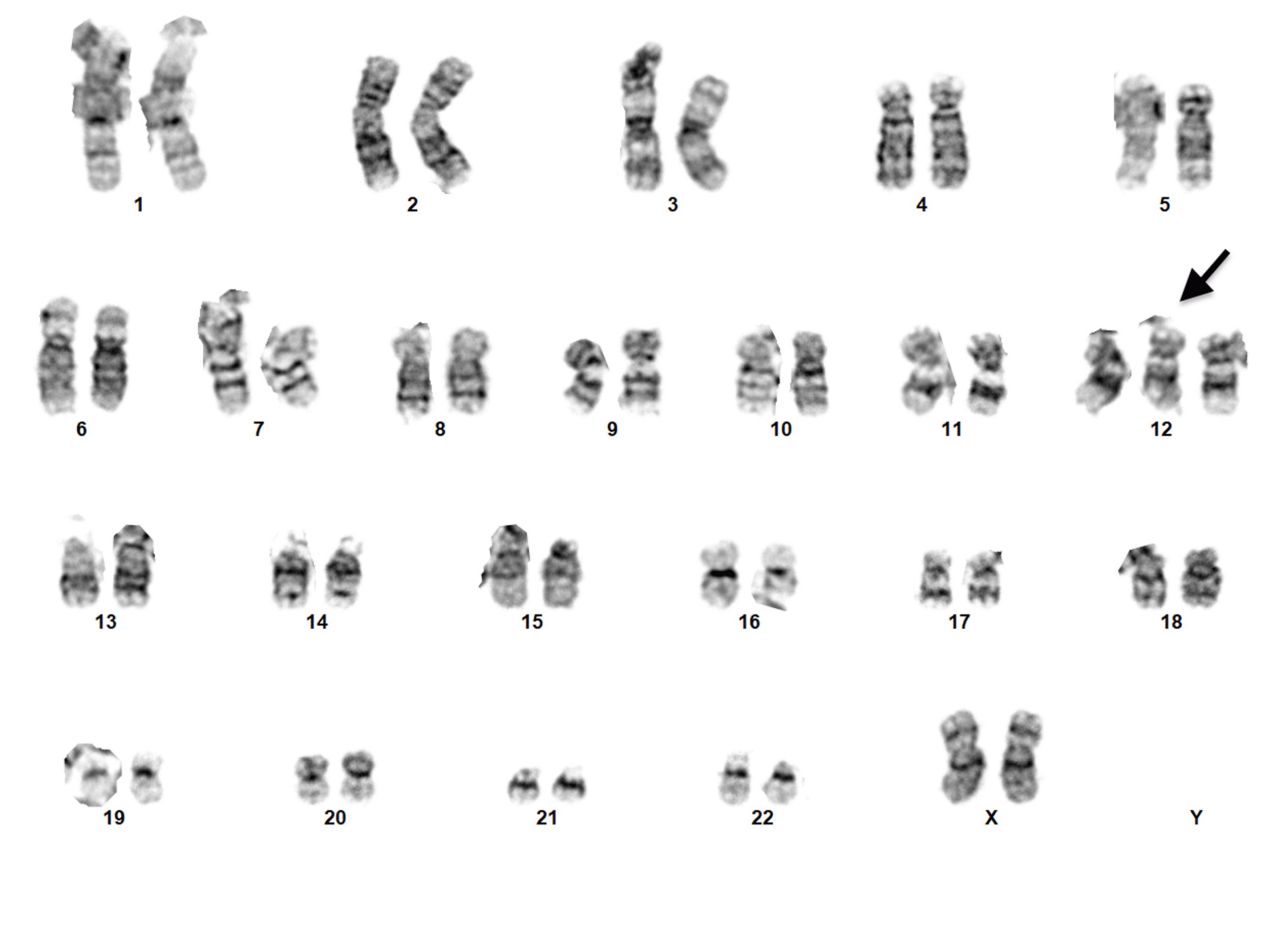
Bone marrow chromosome analysis Chromosome analysis performed on the bone marrow showed gain of chromosome 12 (arrow) in 6/20 (30%) metaphases examined

Chromosome analysis performed on the bone marrow showed a gain of chromosome 12 in 6/20 (30%) metaphases examined (Figure 5). The remaining 14/20 (70%) showed a normal karyotype. The presence of trisomy 12 supported a diagnosis of marrow involvement by CLL/SLL. FISH was performed on the bone marrow and was negative for MYC, BCL6, and IGH::BCL2 gene rearrangements.

Thus, the patient was diagnosed with two separate lymphomas: CLL/SLL, stage IVA, and BCL2-R-negative, CD23+ follicle center lymphoma, stage 2A.

The patient remained on observation for 8 months when she noticed an enlarging right parotid mass along with multiple bilateral cervical lymph nodes. CT revealed enlargement of the right superficial parotid nodule, measuring up to 4.1 cm, a new contralateral left superficial parotid nodule measuring up to 1.4 cm, and multiple heterogeneously enhancing right level 2, 3, and 5 lymph nodes. A biopsy of the right parotid gland mass was performed, revealing findings similar to the previous biopsy - BCL2-R-negative, CD23+ follicle center lymphoma. However, the biopsy sample was very small, and the CLL/SLL component could not be properly appreciated on morphology. The B-cell gene rearrangement study was repeated and showed two separate clones identical to those identified at the time of initial diagnosis. At this time, treatment was initiated with bendamustine and rituximab since this regimen would help both her follicular lymphoma as well as CLL/SLL. She has now completed five of a total of six cycles and is doing well with evidence of clinical response.

## Discussion

Follicular lymphoma (FL) is a neoplasm arising from follicle center cells with at least a partial follicular growth pattern and a recurrent genetic alteration involving t(14:18). However, FL is a heterogeneous disease, and t(14:18) negative variants are now recognized. Approximately 5% of low-grade follicular lymphomas (LGFL) show a diffuse pattern and ~10% lack the BCL2 rearrangement [4]. Other low-grade B-cell lymphomas, such as nodal marginal zone lymphoma and pediatric type follicular lymphoma, must be carefully excluded. BCL2-R-negative, CD23+ follicle center lymphoma cases tend to be more genomically stable than FL. Recurrent genetic mutations and copy number alterations associated with FL can be seen in BCL2-R-neg FL, but at a lower frequency. Activating mutations in STAT6, found in more than half of cases, are among the most common genetic alterations in BCL2-R-negative follicular lymphomas. Additional recurrent mutations affect genes involved in epigenetic regulation, including the histone methyltransferases KMT2D and EZH2, the histone acetyltransferases CREBBP and EP300, and the tumor necrosis factor receptor gene TNFRSF14. Together, these alterations define a molecular profile that is distinct from that of classical BCL2-rearranged follicular lymphoma [4]. Fewer copy number alterations are seen in BCL2-R-neg follicular lymphomas compared to cFL, where more frequent gains in the chromosomal regions 1q, 2p15, 7, 8q, 12q, 18p, and 18q, as well as losses in 1p36, 3q, 6q, 9p, 10q, 11q, 13q, and 17p may be identified. BCL2-R-negative, CD23+ follicle center lymphoma is the only low-grade nodal follicular lymphoma that lacks the IGH::BCL2 gene rearrangement. Katzenberger et al. first outlined this lymphoma subtype in 2009, noting its deviation from conventional follicular lymphoma through features such as a predominantly diffuse architecture, female predominance, inguinal presentation, and generally favorable clinical behavior in early-stage disease [4].

At the molecular level, it is defined by the absence of the IGH::BCL2 fusion and commonly features mutations in STAT6 (57%), which is the most common, and CREBBP (49%), as well as 1p36 deletions or TNFRSF14 (39%) mutations. CD23 expression in follicular center lymphoma without BCL2 rearrangement appears to result from activating STAT6 mutations [9]. CD23 expression has been reported to be a surrogate marker for STAT6 [3]. Although the inguinal lymph nodes are described as the common site of occurrence, our case was unusual in that the lymphoma presented at multiple sites involving the parotid glands, cervical, and axillary lymph nodes. Further, the synchronous occurrence of the CD23+ dFL with chronic lymphocytic leukemia/ small lymphocytic lymphoma (CLL/SLL) made this case complex. The diffuse growth pattern, CD23 expression, and similar light chain expression of the two lymphomas threw up many diagnostic challenges, which required careful morphologic assessment as well as ancillary testing with flow cytometry and genetic studies for accurate diagnosis. Morphologically, the tumor comprised a subset of lymphoid cells with centrocytic morphology that was positive for CD20, PAX5, CD10, BCL6, and CD23, and negative for CD5 and BCL2. A second population of monotonous small lymphocytes surrounded this subset and was present in the interfollicular areas, expressed CD20 (dim), PAX5, and CD5 (weak), and was negative for CD10. Flow cytometry demonstrated two phenotypically distinct B-cell subsets, one being CD5+ kappa-restricted and the other CD10+ kappa-restricted. The BCL2 negativity by IHC using two BCL2 antibodies was confirmed by the absence of IGH::BCL2 rearrangement by FISH. BCL6 rearrangements and 1p36 deletions were also negative by FISH. Next-generation gene sequencing was performed for additional endorsement of the morphologic diagnosis of CD23+ dFL and CLL/SLL due to the distinct molecular profiles of each entity. Mutations involving STAT6 (VAF 5.4%), TNFRSF14 (VAF 5.4%), CREBBP (VAF 8.1%), CREBBP (VAF 6.2%), and FOXO1 start-loss were supportive of the diagnosis of CD23+dFL, while detection of trisomy 12 by FISH was supportive of the diagnosis of CLL/SLL. PCR-based IGH gene rearrangement studies were critical in establishing the clonal relationship between the two lymphomas. Interestingly, the results revealed two separate B-cell clones, a biallelic clone and a second clone, of distinctly different sizes (~360 bp and ~299 bp respectively) in both frameworks 1 and 2 and also the B-cell gene rearrangement study was repeated on her parotid biopsy and showed two separate clones identical to those identified at the time of initial diagnosis. The findings demonstrated that the two lymphomas had arisen from two unrelated B-cell clones, confirming the morphologic diagnosis of two distinct B-cell lymphomas. A large cell transformation of CLL was unlikely given the absence of a large cell expansion. Further, these are clonally related tumors, while in our case, the tumors arose from two unrelated clones. This case underscores the role of flow cytometry, cytogenetics, gene rearrangement studies, and molecular testing in accurately navigating the challenges posed by complex cases.

## Conclusions

Composite lymphomas comprising two clonally unrelated indolent B-cell lymphomas occurring at multiple sites are extremely uncommon. To the best of our knowledge, here we present the first case of a composite lymphoma involving the newly described CD23+ dFL and CLL/SLL. These cases throw up many diagnostic challenges and highlights the importance of detailed workup with morphology, immunophenotyping with immunohistochemistry and flow cytometry, and molecular studies. This case adds to the heterogeneity of BCL2-negative follicular lymphomas and the understanding of the biology and management of these complex cases.
